# Use of Computational Intelligence in Customizing Drug Release from 3D-Printed Products: A Comprehensive Review

**DOI:** 10.3390/pharmaceutics17050551

**Published:** 2025-04-23

**Authors:** Fantahun Molla Kassa, Souha H. Youssef, Yunmei Song, Sanjay Garg

**Affiliations:** Centre for Pharmaceutical Innovation (CPI), Clinical and Health Sciences, University of South Australia, Adelaide, SA 5000, Australia; fantahun.kassa@mymail.unisa.edu.au (F.M.K.); souha.youssef@unisa.edu.au (S.H.Y.); may.song@unisa.edu.au (Y.S.)

**Keywords:** computational intelligence, CI, three-dimensional printing, 3D printing, customizing drug release, personalized medicine, CI-driven drug release

## Abstract

Computational intelligence (CI) mimics human intelligence by expanding the capabilities of machines in data analysis, pattern recognition, and making informed decisions. CI has shown promising contributions to advancements in drug discovery, formulation, and manufacturing. Its ability to analyze vast amounts of patient data and optimize drug formulations by predicting pharmacokinetic and pharmacodynamic responses makes it a very useful platform for personalized medicine. The integration of CI with 3D printing further strengthens this potential, as 3D printing enables the fabrication of personalized medicines with precise doses, controlled-release profiles, and complex formulations. Furthermore, the automated and digital capabilities of 3D printing make it suitable for integration with CI. CI has proven useful in predicting material printability, optimizing drug release rates, designing complex structures, ensuring quality control, and improving manufacturing processes in 3D printing. In the context of customizing drug release from 3D-printed products, CI techniques have been applied to predict drug release from input variables and to design geometries that achieve the desired release profile. This review explores the role of CI in customizing drug release from 3D-printed formulations. It provides overview of limitations of 3D printing; how CI can overcome these challenges, and its potential in customizing drug release; a comparison of CI with other methods of optimization; and real-world examples of CI integration in 3D printing.

## 1. Introduction

CI emulates human intelligence by expanding the capabilities of machines in data analytics, pattern recognition, and making informed decisions [1,2,3]. In the pharmaceutical area, CI has shown promising contributions to advancements in drug discovery, formulation, and manufacturing [4,5]. For example, CI can assist in drug repurposing, where it can predict the interactions of drugs with their targets, optimize molecular structures, and identify clinical trial candidates [6,7,8]. CI is also involved in simplifying supply chain management, prediction of drug efficacy, and adverse drug reaction profiling that speed up drug development [9,10,11].

CI’s ability to analyze vast amounts of patient data and optimize drug formulations by predicting pharmacokinetic and pharmacodynamic responses makes it a very useful platform for personalized medicine [12,13,14,15]. Personalized medicine is an approach that tailors treatments to individual patient needs [16,17,18,19,20]. Unlike the conventional one-size-fits-all approach, which has an estimated success rate of only 30 to 50% [21,22], personalized medicine aims to enhance therapeutic efficiency and minimize adverse drug reactions by considering factors such as age, sex, body mass, comorbidities, and genetic variability [16,23,24].

CI’s potential for personalized medicines cannot be fully realized with traditional large-scale manufacturing as it produces medications in fixed doses that may not align with individual patient needs [25,26,27,28]. The common practice of manual dose adjustments, such as splitting or crushing tablets, can lead to dosing inaccuracies and disrupt controlled-release coatings designed to regulate the active ingredient’s release rate [19,29,30,31]. This gap between personalized treatment and large-scale production highlights the need for more flexible manufacturing methods.

Three-dimensional (3D) printing is emerging as a promising solution to these challenges, offering the flexibility to manufacture personalized medicines with precise doses, controlled-release profiles, and complex formulations [27,32,33]. By enabling on-demand production, 3D printing can allow the fabrication of patient-specific medications, reducing the need for extensive reformulation and ensuring better alignment with individual therapeutic needs. Its ability to create multilayered or compartmentalized dosage forms also holds potential for applications in diseases requiring complex release strategies [19,26,27,34,35].

When integrated with CI, 3D printing has the potential to further optimize drug formulations by leveraging patient-specific data, such as genetic information, disease progression, and metabolic responses [5,21,36]. For example, Fawzi et al. [37] combined CI with a 3D-printed wearable insulin pump for type 1 diabetes mellitus patients, where the CI system calculates individualized doses, wirelessly transmitting them to an Internet of Things (IoT)-enabled pump system to administer the prescribed dosage to the patient. This integration can facilitate on-site drug production in hospitals and clinics, allowing healthcare providers to create patient-specific medications while reducing waiting times and adapting formulations to an individual’s health status [38].

Several studies have investigated the integration of CI in 3D printing to optimize drug release profiles. CI models have been used to predict drug release based on input variables and to design geometries that achieve desired release rates. While there are existing reviews on the integration of CI and 3D printing, they broadly cover CI’s role in various stages of the 3D printing process, such as material selection, printability optimization, quality control measures, and structural design [39,40,41,42]. This review specifically emphasizes on CI’s roles in customizing drug release profiles. It provides an overview of the limitations associated with using 3D printing for drug delivery systems, summarizes how CI has been employed to address these challenges, and discusses CI-driven approaches for tailoring drug release. The review also includes real-world examples of CI integration in 3D printing and compares CI methods with other optimization approaches.

## 2. Limitations of 3D Printing for Drug Delivery Systems Manufacturing

3D printing is a manufacturing technique that uses a layer-by-layer approach to produce 3D objects of the desired size, shape, and structure [43]. The manufacturing process begins with the modelling of an object with computer-aided design (CAD) software, followed by slicing it into layers and finally printing of the designed model [44,45,46,47].

There are multiple 3D printing methods, each offering specific advantages depending on the application. The extrusion, binder jetting, powder bed fusion, vat photopolymerization, and material jetting are commonly employed 3D printing methods in pharmaceutical applications [23]. The working mechanisms and advantages of each were discussed in detail in the literature [22,39,45,48,49,50].

While 3D printing holds considerable promise for pharmaceutical applications, certain challenges limit its application. Material selection is identified as one of the major challenges. Identifying materials that can withstand 3D processing while retaining properties for effective drug delivery is important but remains one of the bottlenecks in advancing 3D printing technology for pharmaceuticals [26,51,52,53].

Another challenge is the complexity of the research and development (R&D) process. Unlike conventional manufacturing, the R&D phase of 3D-printed formulations is highly iterative, with options for heavy customization in most of the technologies in 3D printing. Developers have many factors to consider, from selecting different printing methods to fine-tuning printer settings to meet precise formulation needs. This complex process tends to protract development timelines and increases resource needs, contrary to the pharmaceutical industry’s demand for quick, cost-effective, and sustainable drug production [40].

Quality control (QC) is another challenge for 3D-printed pharmaceuticals. Conventional QC methods are based on sampling from large, uniform batches and are, therefore, not well matched to 3D printers’ small-batch and customized production features [29,50]. Ensuring consistent quality without compromising individual units requires nondestructive QC methods tailored to the unique needs of 3D-printed formulations [40,45]. Recent studies have demonstrated the feasibility of nondestructive QC using process analytical technologies (PATs). For example, near-infrared and Raman spectroscopy have been employed for drug dose determination [54,55,56] and amorphous content quantification [57], while near-infrared spectroscopy alone has shown potential for predicting tablet density and drug release [58]. In addition, a pressure sensor has been integrated into the printer to enable real-time material characterization and monitoring of the printing process [59], and an inbuilt analytical balance has been incorporated for mass uniformity testing [60].

## 3. Role of Computational Intelligence in Addressing Limitations of 3D Printing

To address the limitations of 3D printing, various CI models have been integrated with 3D printing. The automated and digital capabilities of 3D printing make it suitable for such integration [39,61]. CI models have been effectively employed for material selection by predicting printability, optimizing printing parameters, and designing complex structures to achieve targeted drug release profiles. Moreover, these models have been applied in anomaly detection for quality control, forecasting carbon dioxide emissions during the printing process, and generating novel formulations (Table 1).

There are different types of computational intelligence, including machine learning (ML), natural language processing, rule-based expert systems, and robotics [62]. Of these, ML is the most widely utilized in 3D printing applications. ML is a branch of CI that involves the application of sophisticated algorithms to analyze and identify patterns in large and complex datasets [21,63,64,65].

Table 1 summarizes studies that have employed ML in pharmaceutical 3D printing. Various ML models are integrated throughout the entire 3D printing workflow, supporting tasks from formulation design to quality control. For instance, deep-learning models such as U-Net have been used in binder jetting to determine drug distribution within printed tablets, while nnU-Net has been applied to medical image segmentation. In direct ink writing, decision trees (DT) and random forests (RF) have been employed to predict the printability of hydrogel formulations. Within fused deposition modeling (FDM), multiple linear regression (MLR), DT, and support vector machines (SVMs) have been used to predict drug dissolution profiles based on rheological data. In addition, generative adversarial networks (GANs) have been used to generate new formulations for 3D printing, while genetic algorithms (GAs) optimize capsule geometry to achieve desired drug release profiles. For quality control in stereolithography (SLA), machine vision combined with SVMs and other algorithms have been employed for anomaly detection. In selective laser sintering (SLS), various ML techniques, including principal component analysis (PCA), random forests (RF), and SVMs have been utilized to predict the printability of formulations. Artificial neural networks (ANNs) are also widely applied with several 3D printers, such as for designing drug delivery scaffolds, optimizing ibuprofen release in digital light processing (DLP), and predicting geometry classification and surface area-to-volume ratios in FDM.

**Table 1:** Applications of machine-learning techniques in 3D printing of pharmaceuticals.

3D Printer	Models Used in This Study	Purpose of the CI	Reference
Binder jetting	U-Net	Determine drug distribution in printed tablets	[11]
Bioprinter	nnU-Net	Medical image segmentation	[66]
	DT, MLR	Predict shape recovery ratio	[67]
	3D printing and neural network co-modelling	Model the relationship between electric field imaging and electroanatomical features of cochlear implants	[68]
	ANN	Design and optimize pseudo-bone drug delivery scaffold for controlled release of simvastatin	[69]
	Regression neural network, classification neural network, Bayesian optimization	Predict cell viability	[70]
	Hierarchical machine learning	Optimize and predict print outcomes	[71]
	Relative least general generalization algorithm, MLR	Predict printability based on ink composition	[72]
	SVM, convolutional neural network (CNN)	Anomaly detection	[73]
	RF	Predict printability from rheological data	[74]
Direct ink writing printer	DT, RF, DL	Predict printability of hydrogel formulations	[75]
	PCA, linear discriminant analysis (LDA), partial least square (PLS), k-means, density-based spatial clustering of applications with noise, hierarchical clustering, t-distributed stochastic neighbor embedding	Verify drug and dose of orodispersible films	[76]
DLP	CNN	Assess print fidelity and uniformity	[77]
	SVM, multiple regression analysis	Predict drug release	[78]
	ANN	Optimize and predict ibuprofen release	[79]
	Self-organizing maps, generalized regression neural network	Predict the influence of tablet thickness on release rates	[19]
FDM	MLR, DT, SVM, partial least squares	Predict drug dissolution profiles from rheological data	[80]
	Evolutionary algorithm	Identify structures for a prescribed drug release profile	[81]
	GAs	Optimize capsule geometry for desired release profiles	[82]
	GAN, a bag of features	Generate 3D porous structures	[83]
	Multivariate linear regression, k-nearest neighbor, SVM, RF, (traditional) neural networks, DL	Predict key fabrication parameters (e.g., temperature, filament characteristics)	[61]
	ANN	Geometry classification and surface area-to-volume ratio prediction	[84]
	RF, k-nearest neighbor, ANN, SVM, logistic regression	Predict hot melt extrusion temperature, filament properties, printability, dissolution time	[85]
	Stochastic gradient descent, DT, Naïve Bayes (NB), multilayer perceptron, SVM, PCA, CNN	Quality control and anomaly detection for fabricated microneedles	[2]
	Extreme gradient-boosted trees	Predict personalized insulin dosages for IoT-reconfigurable system	[37]
	RF, light gradient-boosting machine, DT, extreme gradient boosted trees, SVR, k-nearest neighbor, kernel ridge regression, multilayer perceptron	Forecast CO_2_ emissions from pharmaceutical FDM printing	[86]
	DT	Relate mechanical properties of filaments to printability	[87]
	Self-organizing maps, multilayer perceptron	Predict drug release properties	[88]
	GAN	Create new formulations for 3D printing	[89]
	ANN, SVM, RF	Predict printability and filament properties	[90]
	PCA	Compare the printability of the polymer and identify the contribution of each mechanical property	[91]
Inkjet printing	ANN, SVM, RF	Develop predictive models for printing outcomes	[92]
SLA	Machine vision, SVM, k-nearest neighbor, logistic regression, DT, RF, gradient-boosting, multilayer perceptron	Anomaly detection for quality control	[38]
Semi-solid extrusion (SSE)	SVM, Gaussian model, DT	Optimizing 3D printing parameters	[93]
SLS	PCA, t-distributed stochastic neighbor embedding, RF, logistic regression, SVM, gradient boosting, extreme gradient boosting, DT, multilayer perceptron, k-nearest neighbor, extremely randomized trees	Predict printability of formulations	[94]
	Deep ensembles, extreme gradient boosting	Predict printability of SLS formulations	[21]
Sprayed Multi Adsorbed Droplet Reposting Technology (SMART)	DT, RF, k-nearest neighbor, light gradient boosting machine, extreme gradient boost	Identify factors and predict drug loading efficiency of microparticles	[95]

## 4. Classification of Machine-Learning Techniques

**Figure 1:**
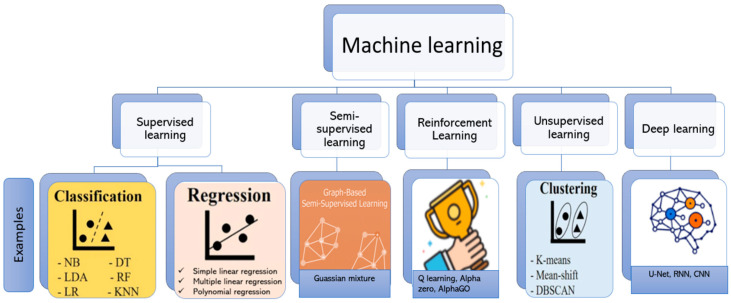
Examples of machine-learning techniques (created with Biorender).

ML techniques are broadly categorized into several approaches (Figure 1), each offering unique methods for data analysis and decision-making [1,4,42,63,96]. A detailed explanation of each approach can be found in the literature [8,9,39].

**Figure 2:**
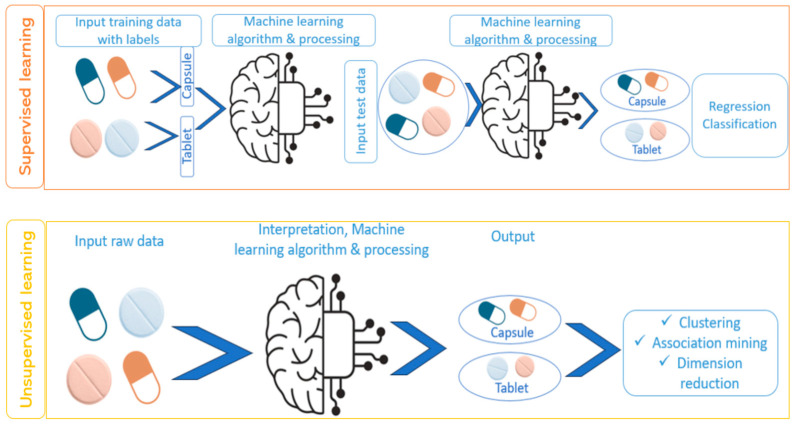
Difference between supervised and unsupervised machine learning (created with Biorender).

In supervised learning, the model is trained on labeled data, where each input is paired with a corresponding output [75]. During training, the model learns the relationship between inputs and outputs, enabling it to predict outcomes for new, unseen data (Figure 2). This approach is commonly employed for tasks such as classification and regression, where precise predictions are required based on historical data [97]. Classification refers to categorizing data into predefined groups or classes based on learned patterns, such as predicting whether a 3D-printed product will meet quality standards (pass/fail) [42]. Conversely, regression involves predicting continuous numerical outcomes, such as estimating the optimal print temperature or the drug release rate from a printed implant [9,39,63]. Unsupervised learning uses unlabeled data—input variables without corresponding outcome variables—and focuses on identifying hidden patterns or underlying structures within the data. It is often applied in clustering and association tasks, such as grouping similar 3D-printed components based on material properties or geometric features (Figure 2) [76].

Semi-supervised learning combines labeled and unlabeled data, which is practical when labeling large datasets is costly. This approach achieves higher accuracy than unsupervised learning alone as there is a small amount of labeled data [36,41].

Reinforcement learning relies on interactions with an environment where rewards or penalties guide the model’s actions, leading it to gradually optimize its behavior through feedback. This approach is beneficial in dynamic systems, such as optimizing 3D printing processes in real time [98].

Deep learning (DL) employs multilayered neural networks to process complex, high-dimensional data. Excelling in areas such as image recognition and natural language processing, DL is an extension of artificial neural networks, characterized by deeper architectures that enable higher-order feature extraction and analysis [39,66,99]. Its capabilities make DL particularly impactful in applications requiring intricate data interpretation, such as analyzing complex 3D models or optimizing drug delivery systems [100].

### 4.1. Implementation of Machine-Learning Techniques

**Figure 3:**
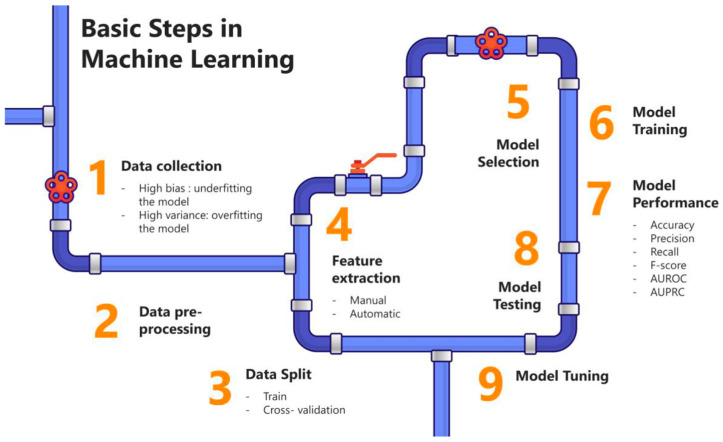
A schematic diagram of the basic steps in machine learning (Adapted from reference [5]). Licensed under a Creative Commons Attribution (CC BY) license (https://creativecommons.org/licenses/by/4.0/, accessed on 13 January 2025).

The implementation of an ML technique involves several structured steps (Figure 3). The process begins with problem definition, where the objective is established, such as predicting drug release profiles or classifying materials. Next, relevant data are gathered from credible sources, including existing literature, experimental results, and clinical datasets [9].

Following data collection, data preprocessing is performed. This step includes data cleaning to handle missing or inconsistent entries, normalization to scale features appropriately, and splitting the dataset into training, validation, and test sets [101].

There are two common techniques for splitting datasets: hold-out validation and cross-validation. The hold-out validation method partitions datasets into a single training and test set, commonly in a 70:30 or 80:20 ratio. In contrast, cross-validation repeatedly divides the dataset into multiple subsets. For example, in k-fold cross-validation, the data are split into k folds, with each fold used as the test set once while the remaining folds serve as the training set [102].

After splitting the data, feature selection is carried out to identify variables that significantly influence the model’s outcomes [5]. Subsequently, an appropriate ML algorithm is selected based on the problem at hand, such as supervised learning for labeled data or unsupervised learning for pattern recognition [85].

The model is trained using the training dataset to learn patterns and relationships within the data. Its performance is assessed using the validation dataset, which is used to fine-tune hyperparameters to improve reliability and accuracy. The test dataset, comprising unseen data, is used to evaluate the final performance of the model objectively [5,42,103].

Model evaluation involves the use of performance metrics, such as accuracy, precision, recall, F1-score, or mean squared error, depending on the problem type. Once the model demonstrates satisfactory performance, it is deployed in real-world applications, such as monitoring drug release in pharmaceutical systems or other relevant tasks.

### 4.2. Evaluation of the Performance of Machine-Learning Models

**Figure 4:**
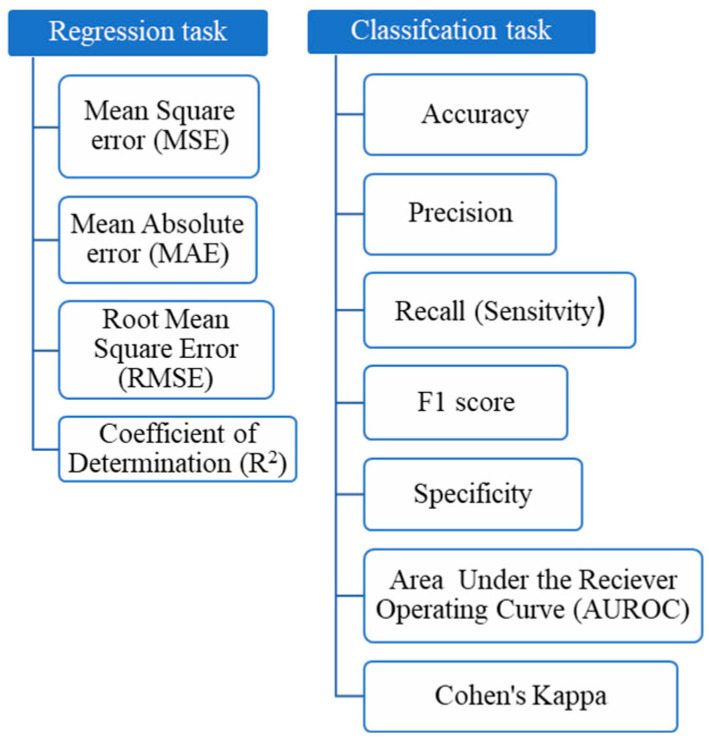
Metrics for evaluating the performance of ML models.

Since no single metric can offer a comprehensive evaluation, a range of metrics is used to assess the performance of ML models [39]. Figure 4 presents the metrics used in regression and classification tasks [3,9,61,85,92], and Table 2 shows the formulas for calculating each metric [104,105,106].

In the regression task, MAE measures the average magnitude of prediction errors and is less sensitive to outliers compared with MSE and RMSE [70]. MSE calculates the average squared difference between predicted and actual values but is more affected by large errors [1]. RMSE, the square root of MSE, shares similar characteristics [95,107] but has the advantage of being expressed in the same unit as the target variable and penalizing large errors more heavily [1]. Since these metrics evaluate model error, smaller values indicate better performance [102]. R^2^, on the other hand, reflects the proportion of variance in the target variable that is accounted for by the model, with values nearing 1 indicating stronger explanatory power [70].

In classification tasks, accuracy represents the proportion of correctly predicted instances among all predictions [21]. While it provides a straightforward measure of overall performance, it does not account for imbalanced datasets [102]. An imbalanced dataset refers to a dataset in which the number of instances (samples) in one class is significantly higher or lower than in the other class(es). This disproportionate class distribution can bias the learning algorithm, often leading to suboptimal performance, particularly in predicting the minority class [108]. Precision evaluates the quality of positive predictions, representing the proportion of correctly identified positives among all predicted positives [70]. Recall (or sensitivity) measures the model’s ability to identify true positive cases, reflecting its effectiveness in detecting all relevant instances [73]. The F1 score combines precision and recall into a single metric, offering a balanced assessment of an ML algorithm’s performance in predicting positive cases [75,94]. On the other hand, specificity measures the proportion of correctly identified negative instances.

The ROC curve and AUC comprehensively evaluate a model’s classification performance across thresholds [5]. The ROC curve plots the true-positive rate (sensitivity) against the false-positive rate (1-specificity) for different classification thresholds. The AUC, calculated as the area under the ROC curve, quantifies performance: a value of 0.5 indicates no discrimination, 0.7–0.8 is considered acceptable, 0.8–0.9 is excellent, and values above 0.9 are outstanding [102].

Cohen’s Kappa measures the agreement between predicted and actual values while accounting for the agreement expected by random chance, making it useful for imbalanced datasets [3]. Kappa values less than 0.4 indicate that the performance is worse than random guessing, values between 0.4 and 0.8 represent moderate performance, and values greater than 0.8 indicate excellent performance [61].

## 5. Machine-Learning Techniques for Customizing Drug Release from 3D-Printed Pharmaceuticals

Customizing drug release plays a crucial role in improving therapeutic outcomes by controlling when, where, and how long a drug is released. This approach helps maintain steady drug levels in the body, reduces side effects by avoiding sharp rises or drops, and allows precise delivery to specific areas, such as tumors in cancer therapy. It also personalizes treatments by tailoring drug release to each patient’s needs, improves convenience with extended-release forms, and aligns drug delivery with the body’s natural rhythms for better outcomes [109,110,111,112,113].

To customize drug release profiles from 3D-printed dosage forms, it is essential to optimize various process and formulation parameters. For instance, the surface area-to-volume (SA/V) ratio, geometry, thickness, infill density, and polymer network structure have been identified as critical factors [114,115,116,117,118,119]. The design of an experiment has been widely used to optimize these parameters due to its structured and efficient approach. However, in recent years, CI techniques, particularly ML, have gained attention due to their ability to handle complex and multidimensional parameters in drug release systems [22,61,120,121]. For example, ML models inspired by biological structures allow researchers to predict and refine device geometry to achieve targeted release profiles [82].

### 5.1. Artificial Neural Networks for Predicting Drug Release Profiles

ANNs are powerful machine-learning models inspired by the human brain’s neural structure. Composed of interconnected nodes or “neurons”, ANNs excel at recognizing complex data patterns, making them particularly suitable for modeling dynamic, nonlinear systems [3].

**Figure 5:**
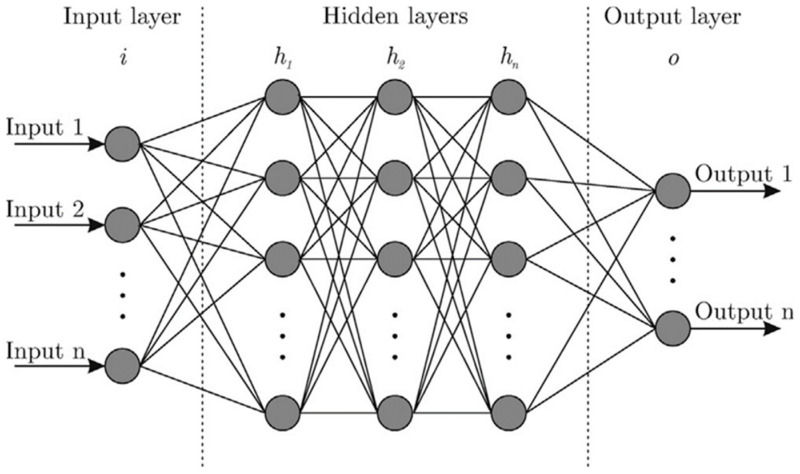
Structure of the layers of artificial neural networks (Adapted from reference [122]. Licensed under a Creative Commons Attribution (CC BY) (https://creativecommons.org/licenses/by/4.0/, accessed on 13 January 2025).

An ANN typically has three main layers: an input layer, one or more hidden layers, and an output layer, as shown in Figure 5 [42]. The input layer’s neurons correspond to the number of process parameters in the study, while the output layer’s neurons represent the number of properties to be optimized, usually one or two. The hidden layer, positioned between the input and output layers, typically has more neurons than the input layer to enable effective pattern recognition and learning [41].

Selecting the appropriate number of neurons in the hidden layer is critical. Too few neurons may lead to underfitting, while too many can cause overfitting, compromising the model’s performance in machine learning [41].

There are different types of ANNs such as multilayer perceptrons (MLPs), conventional ANNs (CANNs), recurrent neural networks (RNNs), and generalized regression neural networks (GRNNs). Each type has distinct applications; for instance, MLPs excel in classification tasks, CANNs are useful for general modelling, and RNNs are preferred for sequential data. ANNs are valued for adapting to dynamic environments, making them ideal for handling nonlinear, complex problems [122].

ANNs have been employed across various aspects of 3D printing. For example, they are used to predict shape recovery ratios [67], optimize 3D-printed pseudo-bone drug delivery scaffolds [69], assess print fidelity, predict printability [21,77,90,92], predict cell viability [70], as well as predict key fabrication parameters such as temperature, filament characteristics, and dissolution rates [61,85]. Additionally, ANNs have been used to forecast carbon dioxide emissions from pharmaceutical FDM printing [86]. In quality control, ANNs play a role in anomaly detection during fabrication, identifying deviations that may impact the final product quality [2,38,73].

Several studies underscore the practical impact of ANNs in predicting drug release profiles based on specific formulation parameters. Castro et al. [85] evaluated five machine-learning techniques, including ANNs, to predict drug dissolution performance, filament mechanical characteristics, and printability from input variables: material details (name, type, and physical properties), surface area, weight, infill density, and the pH of the media. Data for 968 formulations were collected from 114 articles and split into 75% training and 25% test datasets. Among the models tested, the ANN demonstrated the best performance, achieving an MAE of 24.29 min and an R2 value of 0.86, showcasing its strong predictive capabilities for dissolution time.

**Figure 6:**
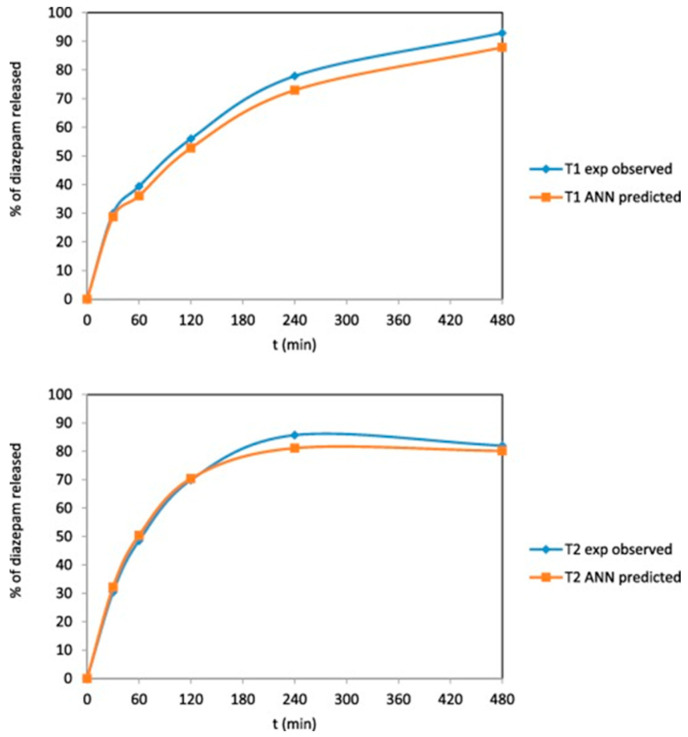
Comparing the experimental dissolution profiles of diazepam in FDM-printed tablets versus predicted (Reproduced with permission from reference [88]. Copyright© 2021 Elsevier).

Obeid et al. [88] developed an ANN model to explore how surface area-to-volume (SA/V) ratio, infill density, and infill pattern influence diazepam release from FDM tablets made from polyvinyl alcohol filaments. Self-organizing maps (SOMs) were used to visualize relationships among input parameters, while an MLP was implemented to predict drug release profiles. The MLP model, structured with 2–3–5 layers, was trained using a back-propagation algorithm over 100 epochs. The dataset was split into training, validation, and test subsets. The MLP achieved RMS error values of 0.143 (training), 0.140 (validation), and 0.093 (test), demonstrating robust predictive performance. The trained model effectively predicted drug release behavior, with similarity factors of 70.24 and 77.44 for two test formulations (Figure 6).

**Figure 7:**
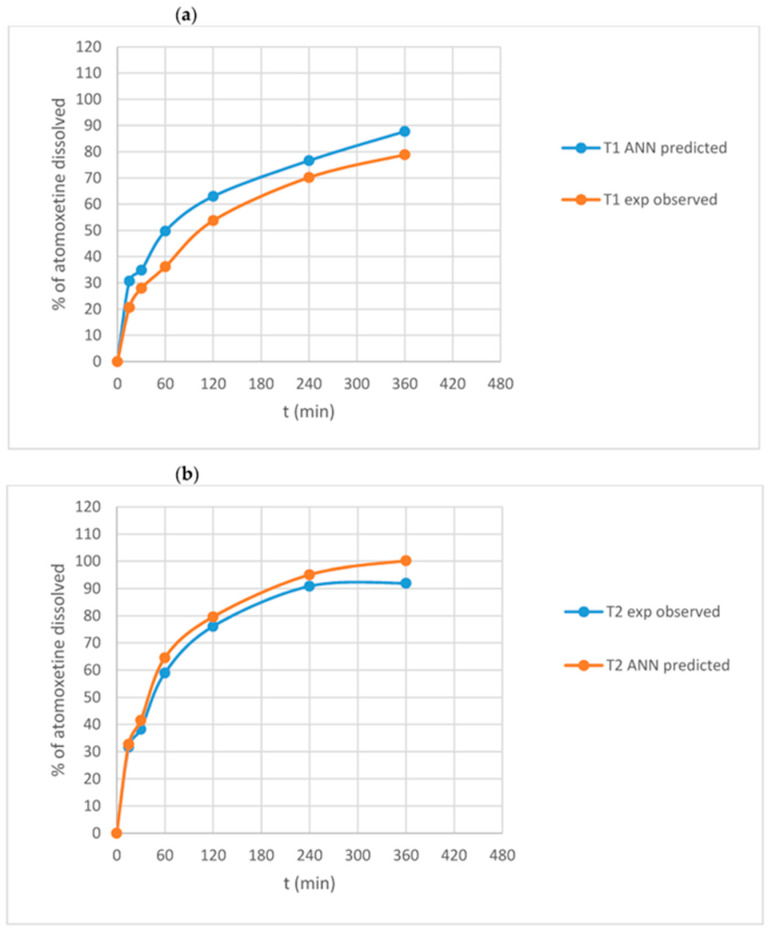
Experimentally observed versus GRNN-predicted dissolution profiles of atomoxetine hydrochloride from formulations (**a**) T1 and (**b**) T2 (Adapted from reference [19]. Licensed under a Creative Commons Attribution (CC BY) (https://creativecommons.org/licenses/by/4.0/, accessed on 13 January 2025).

In another study, Stanojevic et al. [19] developed ANN models to predict atomoxetine hydrochloride release rates from digital DLP 3D-printed tablets. They used SOM for clustering and visualizing experimental data from 23 formulations. A GRNN was trained using 17 experiments, validated with four, and tested with two. The GRNN effectively predicted dissolution profiles, with similarity factors of 51.05 and 70.13 for the test formulations (Figure 7).

Madzarevic et al. [79] investigated the effects of formulation factors on printability and optimized extended drug release from cross-linked polymeric ibuprofen printlets using ANNs developed with two software platforms The input variables included percentages of polyethylene glycol diacrylate, polyethylene glycol, and water. The networks were trained on datasets divided into training, validation, and test subsets. Neural Network 1 achieved an R2 value of 0.9811, while Neural Network 2 achieved an even higher R2 value of 0.9960, both indicating strong predictive accuracy. Furthermore, the dissolution profile predicted by Neural Network 1 closely matched the experimental results, as indicated by a difference factor of 14.30 and a similarity factor of 52.15.

Konidah et al. [69] used an ANN model to optimize a 3D-printed pseudo-bone scaffold for the controlled release of simvastatin. By training the model on 39 formulations with varying concentrations of three polymers, they achieved a controlled release of simvastatin over 20 days.

In contrast to studies that use formulation parameters to predict drug release rates, Mazur et al. [84] explored a reverse approach, applying an ANN model to use drug release profiles for predicting the geometry of 3D-printed dosage forms. The model aimed to optimize key geometric parameters significantly influencing drug release rates. Although direct geometry predictions from dissolution profiles were ineffective, the model demonstrated a strong ability to estimate the SA/V ratio, achieving an MSE of 0.054.

### 5.2. Genetic Algorithms for Optimizing Dosage Form Geometry for Desired Drug Release Profile

GAs are computational methods inspired by natural selection and biological evolution, widely employed for solving complex optimization problems [123,124].

The GA process begins with forming an initial population, where each member represents a potential solution, such as a specific dosage form geometry [7]. These solutions are evaluated using a fitness function, which is problem-specific, for instance, how closely the drug release profile aligns with therapeutic requirements. The fittest solutions are selected as “parents” to generate the next generation through recombination (crossover) and mutation [124]. Recombination combines traits from two-parent solutions, while mutation introduces variability to prevent premature convergence and ensure diverse design space exploration. This iterative process gradually refines the population, yielding optimal or near-optimal [81,123,125].

Genetic algorithms have been applied to solve inverse problems in 3D-printed pharmaceuticals to find the geometry or arrangement within the dosage forms that achieve a desired release profile. For example, a study by Grof and Štepánek [125] employed a GA to configure 3D-printed tablet segments for target dissolution profiles. In their approach, the tablet structure was represented as a sequence of distinct segments, each with defined dissolution rates and active pharmaceutical ingredient concentrations. The GA efficiently identified optimal structural configurations to produce various release profiles, including immediate, delayed, and stepwise drug release.

**Figure 8:**
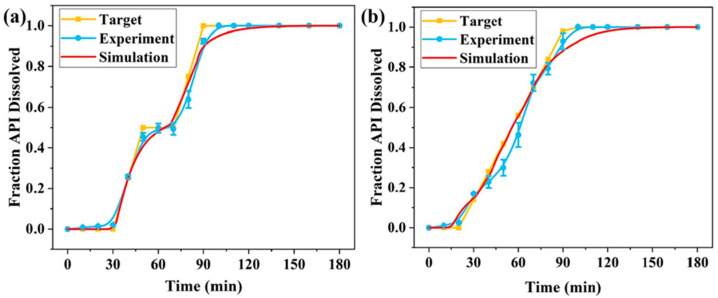
The CI-driven designed capsule with acetaminophen: (**a**) stepwise release; (**b**) uniform release (Reproduced with permission from reference [76] Copyright© 2024 Elsevier).

Another study by Hu et al. [82] applied a genetic algorithm to design multilayered capsules for personalized medicine. By encoding the geometry of capsule layers into a binary sequence, the GA optimized capsule structure to produce controlled drug release profiles. The dissolution process was simulated using the Monte Carlo method to model the random erosion of polymers in aqueous solutions, while the Noyes–Whitney model was applied to describe drug dissolution behavior. The similarity factor was employed to assess the alignment between the simulated and target release profiles. Acetaminophen and isoniazid (200 mg each) were used as model drugs. The results demonstrated the potential of GAs to design complex multilayered structures capable of stepwise or zero-order drug release, with similarity factor values exceeding 50, indicating a good agreement between experimental and simulated release profiles (Figure 8).

The results of the above studies suggest the potential of genetic algorithms in optimizing complex geometric structures to achieve a desired release profile, which contributes to the realization of personalized medicine with 3D printing.

### 5.3. Other Machine-Learning Techniques for Drug Dissolution Prediction

In addition to ANNs and genetic algorithms (GAs), other ML techniques have shown promise in predicting drug release from 3D-printed products. Elbadawi et al. [80] explored various ML methods, including multiple linear regression (MLR), DT, SVM, and partial least squares analysis, to assess the potential of rheological properties, particularly viscosity measurements, in predicting drug release profiles of 3D-printed tablets. Their findings revealed that ML models, particularly the DT model, can accurately predict drug dissolution profiles based on viscosity data. The study concluded that viscosity measurements can be valuable predictors for material printability and drug release profiles in 3D-printed formulations.

**Figure 9:**
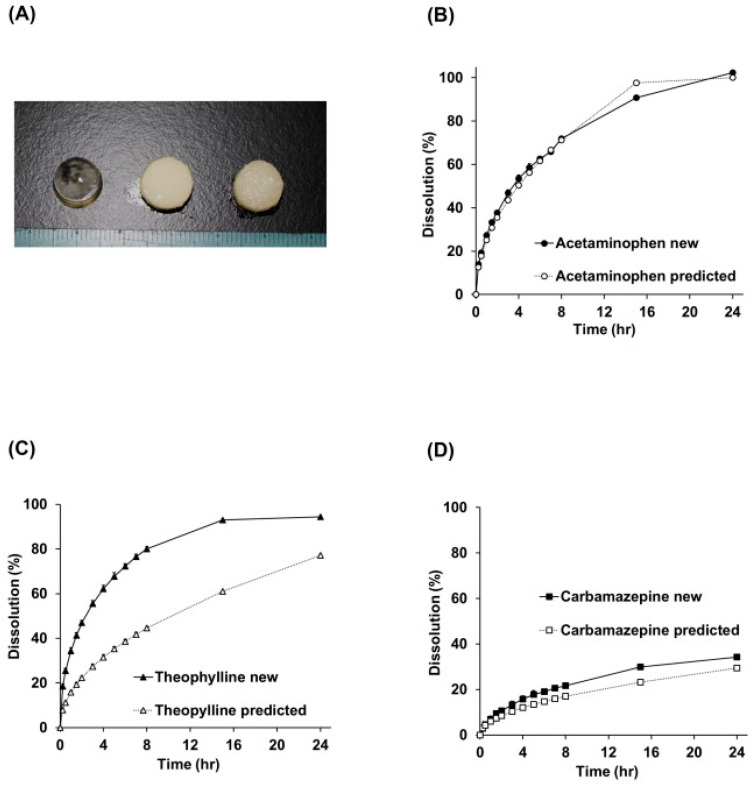
Comparison of actual data and predicted data for drug release from new 3D-printed tablets. (**A**) Appearance of the 3D-printed tablets. (**B**) Drug dissolution profile of acetaminophen. (**C**) Drug dissolution profile of theophylline. (**D**) Drug dissolution profile of carbamazepine (Reproduced with permission from reference [78] Copyright© 2021 Elsevier).

Another study by Tagami et al. [78] developed a predictive model using MLR to estimate the amount of drug released after 15 h. The model incorporated various input variables, including drug type and concentration, poly(ethylene glycol) diacrylate concentration, light exposure time, and water content. To classify the release kinetics based on the predicted drug release amounts, an SVM was employed. The performance of the regression model was assessed using the hold-out validation method and K-fold cross-validation. With a test size of 0.3, the hold-out validation method yielded a high score of 0.944, and K-fold cross-validation also demonstrated robust generalization with a score of 0.933. The study further validated the model by comparing actual and predicted drug release profiles for acetaminophen, carbamazepine, and theophylline. Their results showed that the predicted profiles closely matched the actual release of acetaminophen and carbamazepine, but not for theophylline (Figure 9). According to the authors, the faster-than-predicted actual release of theophylline is likely due to overestimation caused by the limited sample size.

## 6. Real-World Examples of Computational Intelligence Integration in Pharmaceutical 3D Printing

The integration of CI with 3D printing has moved beyond theoretical research into practical applications. One prominent example is M3DISEEN, a CI-empowered platform developed by FabRx, a London-based company, to assess 3D printability in FDM. Initially, the platform was trained using 614 formulations made from 145 different materials, including seven drugs, and employed five ML models to predict extrusion temperature, printing temperature, filament mechanical characteristics, and overall printability [61]. Later, the database was expanded and updated by data mining from 114 published articles and including 968 formulations to the same ML models to predict the aforementioned parameters and drug release rate [85]. Further updates added 1594 formulations from in-house experiments and mined from published articles, and were included to enhance the performance of the models [90]. The platform is freely available online (Home|M3diseen, https://m3diseen.com/home accessed on 12 January 2025) and is a valuable tool for researchers and manufacturers.

FabRx has also developed the first GMP-approved pharmaceutical 3D printer, M3DIMAKER™, available in single and multi-nozzle configurations. Its software, M3DIMAKER Studio, integrated with CI to streamline the process and helps validate the printing process [126]. 

**Figure 10:**
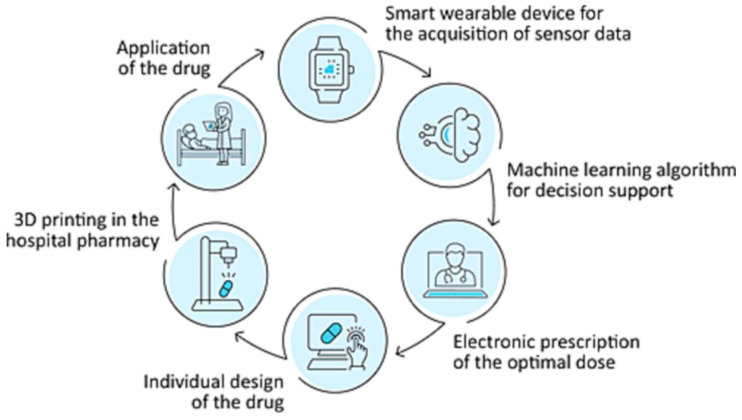
Integration of 3D printing into the digital medication process (Adapted from reference [127]. Licensed under a Creative Commons Attribution (CC BY-NC-ND license) (https://creativecommons.org/licenses/by/4.0/, accessed on 13 January 2025).

CI-driven 3D printing is also undergoing clinical evaluation in hospital settings. A feasibility study by Langebrake et al. [127] aims to integrate CI-assisted 3D printing of medicines into a German hospital’s closed-loop medication management system. The study focuses on Parkison’s patients, who will wear smart wearable devices to collect movement-related data, including tremors, akinesia, and dyskinesia. ML algorithms will analyze these data to determine the optimal drug dosing for levodopa/carbidopa, after which a direct powder extrusion printer will be used to print patient-specific tablets that meet the European Pharmacopeia criteria for solid dosage form (Figure 10).

## 7. Other Methods for Optimizing Drug Release from 3D-Printed Products

In addition to CI models, other computational and statistical methods such as design of experiments (DoE), finite-element analysis (FEA), and mechanism-based kinetic modeling have been used to optimize drug release from pharmaceutical products.

### 7.1. Design of Experiments

DoE is a widely used statistical method for optimizing multiple variables by assessing the impact of various input factors on a target response [39]. The process includes defining objectives, identifying critical variables, designing experiments, constructing mathematical models, analyzing data, and validating results [128]. Unlike the traditional one-variable-at-a-time approach, which is often inefficient and time-consuming, DoE enables the simultaneous evaluation of multiple factors and their interactions [129]. Common DoE techniques, such as factorial designs, response surface methodology (RSM), D-optimal mixture design, and Taguchi methods, facilitate experimental optimization, improve predictability, and enhance the efficiency of formulation development [121,130]. Regulatory agencies such as the FDA emphasize DoE within quality by design (QbD) frameworks to enhance drug product consistency and efficacy [129].

In optimizing drug release from 3D-printed drug delivery systems, DoE has been used to optimize critical parameters such as infill percentage, nozzle temperature, and layer thickness to achieve precise and controlled drug release [131]. For instance, a study by Jeong et al. [132] applied a 2-level factorial DoE to optimize a 3D-printed gastroretentive sustained-release drug delivery system. The effects of the hole size, number, and height on drug release time (T80) were systematically analyzed, leading to the development of a predictive model. The study found that the hole height had the most significant impact on T80, while the hole size and number also contributed to variations in drug release. The model enables a good prediction for the design of fast-, medium-, and slow-release profiles with an MAE ≤ 15.3%.

### 7.2. Finite-Element Analysis

FEA is a numerical method that discretizes a complex structure into small elements and solves partial differential equations to model diffusion, mass transfer, and mechanical behavior [128,133,134]. Unlike traditional empirical drug release studies, which heavily rely on experimental trial-and-error, FEA allows researchers to predict drug release profiles before fabricating the device, reducing material waste and development time [135].

In 3D-printed pharmaceutical applications, FEA has been used to simulate the insertion process of 3D-printed microneedles into the human skin [136,137], evaluate mechanical properties of scaffolds under compressive deformation [138], study the behavior of an optimal elastic artificial disk under different loads for the lumbar spine [139], predict deformations during the 3D printing process of hydrogel scaffolds [140], simulate the mechanical behavior of 3D-printed cochlear implants [141], evaluate the stress–strain response of the 3D-printed suppositories [142], design and optimize a cymbal microneedle array for enhanced transdermal drug delivery [143], and simulate the release behavior of capsules [82].

A study by Haring et al. [144] applied FEA to model the changes in programmed concentration profiles of 3D-printed polypills containing metformin hydrochloride, glyburide, and acarbose due to diffusion during both the printing (processing) and solidification (post-processing) intervals. The study evaluated three polypill designs: core-shell structures, multilayer distributions, and gradient structures. The FEA prediction indicated that the diffusion effect was more pronounced during the post-processing interval than during processing, with the largest deviation occurring in core-shell and multilayer structures, while the gradient structure exhibited smaller changes. These diffusion-driven alterations influenced drug release profiles, leading to distinct release patterns: core-shell designs resulted in delayed release, multilayer structures produced pulsed release, and gradient distributions provided a constant release.

### 7.3. Mechanism-Based Kinetic Modeling

Mechanism-based kinetic models, such as the Higuchi and Korsmeyer–Peppas models, are widely used to describe drug release mechanisms from various delivery systems, including 3D-printed devices. These models are based on the assumption that drug release follows certain physical principles, such as diffusion or swelling, and they provide mathematical expressions to quantify the release rate over time [145].

## 8. Comparison of Computational Intelligence with Other Methods for Optimizing Drug Release

Each approach, DOE, FEA, mechanism-based kinetic modeling, and CI, offers unique advantages but also faces inherent limitations when applied in isolation (Table 3) [39,141,146].

Compared with other optimization methods, CI stands out for its ability to handle complex, nonlinear relationships and integrate vast amounts of data [61]. Furthermore, CI models can adapt and improve over time as new data become available, making them ideal for real-time optimization.

Although CI excels at predicting nonlinear relationships in pharmaceutical formulations, it has limitations, including its “black-box” nature and reliance on large datasets. To overcome these challenges, hybrid optimization approaches have been explored. For example, Madzarevic et al. [79] integrated DoE and CI to optimize ibuprofen release from 3D-printed printlets. A D-optimal mixture design was used to systematically evaluate the effects of polymers and water concentrations on drug release. DoE minimized experimental trials while capturing key interactions between excipients and drug release behavior. The dataset generated from DoE was then used to train an ANN model, enabling it to learn complex, nonlinear relationships between formulation variables and dissolution profiles.

## 9. Conclusions and Future Perspective

The integration of CI, especially ML, has shown great potential in advancing 3D printing for drug delivery applications. CI-driven approaches have proven useful in predicting material printability, optimizing drug release rates, designing complex structures, ensuring quality control, and improving manufacturing processes. In the context of drug release optimization, ML techniques have been applied to predict drug release from input variables and to design geometries that achieve the desired release profile. Methods like ANNs, GAs, and DT have been found effective for this purpose. Among these, GAs and ANNs stand out for their ability to tailor 3D-printed drug delivery systems to meet specific therapeutic needs. However, further research is required to expand their applicability across a broader range of drugs, excipients, and formulation strategies. Additionally, addressing inverse problems, where the desired drug release profile dictates the design parameters, remains an important area of investigation.

Despite these advancements, several challenges must be overcome to fully leverage CI in customized drug delivery. One key limitation is the requirement for large and diverse datasets to effectively train CI models. However, obtaining extensive experimental data is time-intensive and costly, particularly in emerging fields such as 3D-printed pharmaceuticals. There have been efforts to solve the limited availability of data by mining from published scientific articles [9,85]. While efforts have been made to extract data from published literature, issues such as positive result bias and inconsistencies in reported findings hinder data reliability [9]. Recent studies have explored the use of synthetic datasets to supplement experimental data. For instance, Sun et al. [38] employed synthetic images to train several machine-learning techniques for recognizing the quality of tablets, capsules, and film dosage forms produced by the SLA printer.

Another critical challenge is bias in CI training datasets and algorithms, which can compromise prediction accuracy and fairness. Mitigation strategies, such as robust data preprocessing, are essential to enhance model reliability [39,147,148]. Additionally, the “black-box” nature of many CI models limits interpretability, making it difficult for researchers and regulatory bodies to validate predictions [8,9,40]. Explainable CI techniques, which explain the reason behind the decision made by the CI models, may help improve transparency and facilitate broader acceptance [149,150]. Furthermore, the computational demands of DL models pose financial and technical barriers, requiring high-performance computing infrastructure [151].

Beyond technical challenges, ethical and regulatory considerations also play a crucial role in the adoption of CI-driven 3D printing in pharmaceuticals. Privacy concerns regarding patient data, particularly issues related to data ownership and consent, must be addressed through stringent ethical guidelines. Additionally, professional liability remains a significant concern, as failures in CI-driven decision-making or 3D-printed formulations can have serious clinical consequences [39]. Establishing clear regulatory frameworks for CI-assisted 3D printing will be essential to ensure safety, efficacy, and reproducibility in pharmaceutical applications.

Looking ahead, integrating CI with 3D printing has the potential to revolutionize personalized medicine by enabling the development of patient-specific drug delivery systems with tailored release kinetics. However, interdisciplinary collaboration between pharmaceutical scientists, CI researchers, and regulatory authorities will be crucial to overcoming existing challenges and unlocking the full potential of this technology.

## Figures and Tables

**Table 2:** Formulas for calculating common metrics for ML model evaluation.

Metric	Formula
MAE	$\frac{1}{n}\sum_{i=1}^{n}\left|y_{i}^{obs}-y_{i}^{pred}\right|$
MSE	$\frac{1}{n}\sum_{i=1}^{n}{\left(y_{i}^{obs}-y_{i}^{pred}\right)}^{2}$
RMSE	$\sqrt{\frac{1}{n}\;\sum_{i=1}^{n}{\left(y_{i}^{obs}-y_{i}^{pred}\right)}^{2}}$
R^2^	$1-\frac{\sum_{i=1}^{n}{\left(y_{i}^{obs}-y_{i}^{pred}\right)}^{2}}{\sum_{i=1}^{n}{\left(y_{i}^{obs}-y^{obs,mean}\right)}^{2}}$
Accuracy	$\frac{TP+TN}{TP+TN+FP+FN}$
Precision	$\frac{TP}{TP+FP}$
Recall (Sensitivity)	$\frac{TP}{TP+FN}$
F1 Score	$2\times \frac{Precision\times Recall}{Precision+Recall}$
Specificity	$\frac{TN}{TN+FP}$
Cohen’s Kappa	$\frac{P_{0}-P_{e}}{1-P_{e}}$

*y_i_^obs^*: actual observed values, *y_i_^pred^*: predicted values, *TP*: true positive, *TN*: true negative, *FP*: false positive, *FN*: false negative, *P*_0_: observed agreement, *P_e_*: expected agreement by chance.

**Table 3:** Comparison of CI and other methods for optimizing drug release.

Aspect	DoE	FEA	Mechanism-Based Models	CI
Main purpose	Identifies key factors affecting drug release through structured experiments	Simulates solid material behavior, such as drug diffusion and polymer degradation	Uses mathematical equations to describe drug release kinetics based on physical and chemical principles	Uses ML to predict and optimize drug release
Approach	Statistical method using structured experimental plans	Solves partial differential equations to model drug release from solid matrices	Uses established kinetic models (e.g., Higuchi, Korsmeyer–Peppas) to describe drug release profiles	Learns from experimental and simulation data to make predictions and optimize formulations
Data requirement	Requires experimental data but minimizes the number of tests needed	Requires material properties and boundary conditions for accurate simulations	Requires drug release data to fit parameters of kinetic models	Requires large datasets for training predictive models
Strength	- Efficient in identifying key formulation and process parameters - Reduces trial-and-error in experiments	- Provides spatial insights into drug diffusion and polymer degradation - Suitable for complex solid structures	- Simple and widely used in pharmaceutical sciences - Provides interpretable and mechanistic insights into drug release	- Can simultaneously integrate multiple factors - Fast optimization and prediction of drug release based on experimental or simulated data
Limitations	- Lacks mechanistic insights - Limited to the factors included in the experiment	- Requires high computational power - Needs precise material property data	- Oversimplifies complex drug release mechanisms - May not account for dynamic or multiscale processes	- Requires large datasets for accuracy - “Black-box” nature makes interpretation difficult
